# Breast Cancer Stem-Like Cells Are Inhibited by Diosgenin, a Steroidal Saponin, by the Attenuation of the Wnt β-Catenin Signaling via the Wnt Antagonist Secreted Frizzled Related Protein-4

**DOI:** 10.3389/fphar.2017.00124

**Published:** 2017-03-20

**Authors:** G. Bhuvanalakshmi, Basappa, Kanchugarakoppal S. Rangappa, Arun Dharmarajan, Gautam Sethi, Alan P. Kumar, Sudha Warrier

**Affiliations:** ^1^Division of Cancer Stem Cells and Cardiovascular Regeneration, Manipal School of Regenerative Medicine, Manipal UniversityBangalore, India; ^2^Laboratory of Chemical Biology, Department of Chemistry, Bangalore UniversityBangalore, India; ^3^Department of Studies in Chemistry, University of MysoreMysore, India; ^4^Stem Cell and Cancer Biology Laboratory, School of Biomedical Sciences, Curtin Health Innovation Research Institute, Curtin University, PerthWA, Australia; ^5^Department of Pharmacology, Yong Loo Lin School of Medicine, National University of SingaporeSingapore, Singapore; ^6^Cancer Science Institute of Singapore, National University of SingaporeSingapore, Singapore; ^7^Curtin Medical School, Faculty of Health Sciences, Curtin University, PerthWA, Australia; ^8^National University Cancer Institute, National University Health SystemSingapore, Singapore; ^9^Department of Biological Sciences, University of North Texas, DentonTX, USA; ^10^Manipal School of Regenerative Medicine, Manipal UniversityBangalore, India; ^11^School of Biomedical Sciences, Curtin Health Innovation Research Institute, Curtin University, PerthWA, Australia

**Keywords:** breast cancer stem cells, diosgenin, Wnt antagonists, sFRP4, EMT

## Abstract

**Background:** Identification of breast cancer stem cells as the chemo-resistant and tumor-initiating population represents an important milestone in approaching anticancer therapies. Targeting this minor subpopulation of chemo- and radio-resistant stem-like cells, termed as the cancer stem cells (CSCs) and their eradication could significantly enhance clinical outcomes. Most of the presently administered chemotherapeutics target the tumor bulk but are ineffective against the CSCs. We report here that diosgenin (DG), a naturally occurring steroidal saponin, could effectively inhibit CSCs from three breast cancer cell lines, MCF7, T47D and MDA-MB-231, by inducing apoptosis and inhibiting the CSC associated phenotypes.

**Methods:** CSCs were enriched in these cells lines, characterized for CSC traits by immunocytochemistry and flow cytometry. Proliferation and apoptosis assays were performed in these breast CSCs in the presence of DG to obtain the inhibitory concentration. Apoptosis was confirmed with gene expression analysis, Western blotting and propidium iodide staining. TCF-LEF reporter assay, sFRP overexpression and RNAi silencing studies were performed to study regulation of the Wnt pathway. Statistical significance was evaluated by a two-sided Student’s *t*-test.

**Results:** Using the TCF-LEF reporter system, we show the effect of DG on CSCs is predominantly through the network regulating CSC self renewal, the Wnt β-catenin pathway. Specifically, the Wnt antagonist, the secreted frizzled related protein 4, (sFRP4), had a defining role in the action of DG. Gain-of-function of sFRP4 in CSCs could improve the response to DG wherein CSC mediators were inhibited, β-catenin was down regulated and the effectors of epithelial to mesenchymal transition and pro-invasive markers were repressed. Conversely, the loss-of-function of sFRP4 had a reverse effect on the CSC population which therein became enriched, their response to DG treatment was modest, β-catenin levels increased, GSK3β expression decreased and the expression of epithelial markers of CSC was completely abrogated.

**Conclusion:** These findings demonstrate the effect of DG on inhibiting the resilient breast CSCs which could provide a benchmark for the development of DG-based therapies in breast cancer treatment.

## Introduction

Breast cancer is a leading cause of mortality in women worldwide. Emerging data suggest that the root of the origin of tumor is a small subset of cells, distinct from the tumor bulk cells, designated as the cancer stem cells (CSCs). The existence of CD44+, CD24-/low, Lin negative breast cancer stem cells (bCSC) was first revealed by Al-Hajj et al. (2003). Identification of bCSC within the tumors has created a paradigm shift in the focus of cancer therapy and research. Conventional cancer therapies are effective in debulking breast tumors but often fail to eradicate the CSC population. This effect, along with their toxicity on normal tissues, has favored the use of natural phytochemicals over synthetic oncotherapeutics. Curcumin, a dietary polyphenol, has been shown to inhibit bCSC by blocking self-renewal (Kakarala et al., 2010) and epithelial to mesenchymal transition (EMT) (Mukherjee et al., 2014). Similarly, piperine inhibited bCSC self-renewal without causing toxicity to differentiated cells (Kakarala et al., 2010). Diosgenin (DG), a steroidal saponin, occurs in many dietary plants such as fenugreek and wild yam and has been shown to be antitumorigenic in many tumors including hepatocellular carcinoma (Li et al., 2010), breast cancer (Srinivasan et al., 2009), colorectal cancer (Wang et al., 2004), osteosarcoma (Corbiere et al., 2003) and leukemia (Liu et al., 2005). The anti-tumorigenic effect of DG has been shown to be mediated through the STAT pathway (Li et al., 2010), activation of p53 and caspase 3 (Liu et al., 2005) and via the induction of TRAIL death receptor DR5 (Lepage et al., 2011). DG is a naturally occurring phytoestrogen extracted from a variety of roots and tuber and is the bioactive compound of major pulses and edible roots (Deshpande and Bhalsing, 2014). Although DG has been demonstrated to have a tumorigenic effect across many cancers, it has no known toxic effect on non-tumorigenic differentiated cells. Based on the easy availability and occurrence in several natural food sources (Huang et al., 2010), and the documented ability to inhibit several tumors, we proposed to study the effect of DG to inhibit the most tumorigenic population within the cancer, namely the CSCs.

Cancer stem cells being a highly chemo-resistant population, are not only left untouched by chemotherapeutics but the rapid depletion of the non-CSC tumor mass enriches the cell number of the CSCs. Specific targeting of CSCs is therefore imperative for effective clinical outcome of the highly aggressive and metastatic breast cancer. Although several studies have elucidated a potent anti-tumorigenic property of DG, there have been no reports on the potential of DG to destroy CSCs. We explored the effect of DG on breast cancer stem-like cells from three breast cancer lines having differential hormone receptor expression, that is MDA-MB-231, (a triple receptor namely, estrogen receptor-ER), progesterone receptor-PR and human epithelial growth factor receptor2-HER2 negative cell line, T47D (PR and HER2 negative, ER positive) and MCF7 (HER2 negative, ER/PR positive), so that the effect of DG in different types of breast cancers could be analyzed.

We hypothesized that the effect of DG on CSCs could be through targeting the prominent regulatory network of breast CSCs, the Wnt β-catenin pathway. We assessed the effect of this pathway at various levels and found a pronounced presence of Wnt antagonist, secreted frizzled related protein 4 (sFRP4) in mediating DG-induced apoptosis of bCSCs, interlinking with the expression of CSC-specific, proliferation and metastatic markers and the EMT phenotype. Moreover, we also observed a simulated interaction between β-catenin and DG by molecular docking. These studies provide for the first time molecular insights into the action of DG via the Wnt β-catenin network, which corroborates DG as a potential therapeutic strategy for breast cancer treatment.

## Materials and Methods

### Cell Culture, Proliferation and Apoptosis Assays

Three breast cancer cell lines MDA-MB-231, T47D and MCF7 (NCCS, Pune, India) were enriched for bCSCs by growing as mammospheres in growth factor enriched serum-free medium (SFM) as reported previously (Bhuvanalakshmi et al., 2015) in basal medium (DMEM/F-12 + DMEM-HG) with 100 U/mL PenStrep, 2mM GlutaMAX, and containing 20 ng/mL each of epidermal growth factor (EGF), basic fibroblast growth factor (bFGF; R&D Systems), and leukemia inhibitory factor (LIF; Chemicon). All cells were cultured at 37°C in a humid incubator with 5% CO2. MTT was performed as described previously (Bhuvanalakshmi et al., 2015) using DG (Sigma–Aldrich, St. Louis, MO, USA) at concentrations 0–500 μM. After determining the IC50 value at 400 μM after 24 h, the subsequent experiments were at this concentration of DG for 24 h. BrdU and caspase assays were performed as described previously (Bhuvanalakshmi et al., 2014) with bCSCs from MDA-MB-231 and T47D. Intercellular ROS assay was performed by using a Fluorometric Intracellular ROS Kit (Sigma–Aldrich, St Louis, MO, USA) following the manufacturer’s instructions.

### Immunofluorescent Staining

Immunocytochemistry was performed as described previously (Bhuvanalakshmi et al., 2014) for identifying CSCs using CD44 marker, and β-catenin in sFRP4-overexpressed (OE) cells using FITC labeled mouse anti-human antibodies. CD44 marker was characterized in MDA-MB-231 CSCs, before and after DG treatment. Expression of β-catenin was analyzed in MDA-MB-231 cells over-expressing sFRP4, before and after DG treatment and compared with cells containing empty plasmid.

### Flow Cytometry

The percentage of CSCs expressing CD44 was determined by flow cytometry to study the decrease of CD44 after treatment with DG. After drug treatment, the cells were fixed and the CD44 positive population was determined by incubation with mouse anti-human FITC labeled antibodies against CD44 (1:100 dilution, BD Biosciences) as described previously (Bhuvanalakshmi et al., 2015).

### qRT-PCR

These analyses were performed as described earlier (Warrier et al., 2014) for the genes (all primers were purchased from Sigma Aldrich) for CSC, drug resistance, Wnt, apoptosis (primer details indicated in **Table 1**).

**Table 1:** Primer sequences.

Genes	Primers	Base pair	Annealing temperature °C
*CD44*	F-5′-CATCTACCCCAGCAACCCTA-3′	271	56
	R-5′-GGTTGTGTTTGCTCCACCTT-3′		

*CD24*	F-5′-AACTAATGCCACCACCAAGG-3′	188	55
	R-5′-CCTGTTTTTCCTTGCCACAT-3′		

*ALDH*	F-5′-AAAGTCAAAGGCTTCCTGCCC-3′	191	60
	R-5′-CTCCTGGAACACAGGTGACT-3′		

*Ki67*	F-5-CTGCTTTGGGGACTTGACG-3′	201	62
	R-5-GTCGACCCCGCTCCTTTT-3′		

*Cyclin D1*	F-5′-AACTACCTGGACCGCTTCCT-3′	187	61
	R-5′-CCACTTGAGCTTGTTCACCA-3′		

*Bax*	F-5′-GCTGGACATTGGACTTCCTC-3′	247	61
	R-5′-TCAGCCCATCTTCTTCCAGA-3′		

*ABCC2*	F-5′-AGAGCTGGCCCTTGTACTCA-3′	492	60
	R-5′-TGCGTTTCAAACTTGCTCAC-3′		

*sFRP4*	F-5′-CGATCGGTGCAAGTGTAAA-3′	180	60
	R-5′-GACTTGAGTTCGAGGGATGG-3′		

*DKK1*	F-5′-TCCGAGGAGAAATTGAGGAA-3′	157	59
	R-5′-CCTGAGGCACAGTCTGATGA-3′		

*β-Catenin*	F-5′-CGTCCACAACACTCTGGCTA-3′	159	55
	R-5′-GCCAGCACTTCACTGCAATA-3′		

*GSK3-β*	F-5′-ACTCCAGTGGCGAGAAGAAA-3′	241	55
	R-5′-TTGAGGACAGCAGTGTCAGG-3′		

*Twist*	F-5′-CAGCGCACCCAGTCGCTGAA-3′	438	53
	R-5′-CGCCCCACGCCCTGTTTCTT-3′		

*Snail*	F-5′-GAGGCGGTGGCAGACTAG-3′	159	60
	R-5′-GACACATCGGTCAGACCAG-3′		

*N-cadherin*	F-5′-CTCCTATGAGTGGAACAGGAACG-3′	121	63
	R-5′-TTGGATCAATGTCATAATCAAGTGCTGTA-3′		

*E-cadherin*	F-5′-ATTCTGATTCTGCTGCTCTTG-3′	400	60
	R-5′-AGTAGTCATAGTCCTGGTCTT-3′		

*GAPDH*	F-5′-CAGAACATCATCCCTGCATCCACT-3′	181	61
	R-5′-GTTGCTGTTGAAGTCACAGGAGAC-3′		

*RNAi sFRP*4	F-5′-CCATTTGCACCCTGGAGTTC-3′	201	56
	R-5′-TCCACTTAACATCCTCCGGG-3′		

*RNAi sFRP4 T7 promotor*	F-5′-TAATACGACTCACTATAGGGAGA CCATTTGCACCCTGGAGTTC-3′	246	66
	R-5′-TAATACGACTCACTATAGGGAGA TCCACTTAACATCCTCCGGG-3′		


### Western Blot

Western blotting was performed for β-catenin and phospho β-catenin as previously described Bhuvanalakshmi et al. (2014).

### Propidium Iodide Staining

Cancer stem cells with and without DG treatment were incubated with 5 μg/ml propidium iodide (PI) and monitored every 4 h to assess for PI accumulation inside nucleus. Red fluorescence was visualized and captured by using Nikon Eclipse TE2000-U fluorescent microscope. After 24 h, drug treated CSCs and control CSC were subjected to flow cytometry.

### TCF-LEF Reporter Assay

TCF/ LEF plasmid were transfected in MDA-MB-231 cells as per Cignal TCF/LEF Reporter (GFP) Kit (Qiagen) protocol for 24 h using lipofectamine 3000 (Invitrogen). After transfection, cells were treated with TCF/LEF activator Wnt3a for 24 h. TCF/LEF expression activation were confirmed by GFP florescence. Then cells were treated with DG for 24 h and GFP fluorescence was monitored using fluorescence microscopy and flow cytometry.

### sFRP4 Overexpression Studies

sFRP4 gene inserted in plasmid pEGFP N1 (ClonTechIn) was used for over-expressing sFRP4. MDA-MB-231 cells were transfected using lipofectamine 3000 (Invitrogen) with MEM-reduced serum media and without antibiotic for 24–48 h. Efficacy of transfection was confirmed by GFP expression using a fluorescence microscope and by RT-PCR analysis for sFRP4 expression. The GFP positive sFRP4 overexpressing (OE) cells were used for further analysis.

### Angiogenesis Assay

The ability of sFRP4 OE cells to form capillary tubes after treatment with DG was investigated using an *in vitro* angiogenesis assay kit (Millipore, USA) following the manufacturer’s instructions.

### Migration Assay

To study the effect of DG on sFRP4 OE cells, cell migration was analyzed using a Transwell Migration System (BD Biosciences) as described previously (Bhuvanalakshmi et al., 2014, 2015).

### sFRP4 RNAi Synthesis

Total RNA were extracted from sFRP4 over expressed MDAMB cells using the RNeasy Plus Mini Kit (Qiagen) and 1 μg of RNA was reverse transcribed according to RevertAid^TM^ First strand cDNA synthesis Kit (Thermo Scientific) protocol. sFRP4 double standard RNA (dsRNA) of 210 bp was prepared using MEGAscript^®^ RNAi Kit (Life Technology). T7 promoter sequence was synthesized along with sFRP4 primer containing MDA-MB OE sFRP4 cDNA so as to obtain a sFRP4 PCR product with T7 promoter hanging ends (primer details indicated in **Table 1**). From this 1 μg was used to synthesize dsRNA using T7 RNA polymerases as per MEGAscript RNAi Kit (Life Technology) protocol. Synthesized dsRNAs for sFRP4 was dissolved in nuclease free H_2_0 and dsRNAs with respective base pairs was confirmed by running on 1% agarose gel and this sFRP4 RNAi was used for silencing.

### sFRP4 Silencing Study

MDA-MB-231 cells were transfected with 1 μg RNAi using Lipofectamine 3000 (Invitrogen) with MEM-reduced serum media without antibiotic for 3 days. The mRNA levels of sFRP4 silencing was analyzed through gene expression study using RT & qRT-PCR.

### *In vivo* CAM Assay

Chick chorioallantoic membrane (CAM) model was used to analyze the *in vivo* anti-angiogenic property of DG. Embryonated eggs of days 4–5 were procured from Veterinary College, Bangalore, India, after approval by the Institutional Ethics Committee. CSCs of MDA-MB-231 cells, and MCF7 cells without or with DG treatment (400 μM of DG for 24 h), equal cell numbers (2 × 10^6^) in 100 μL of medium (1:1 of CSC medium and Matrigel) were injected in the air sac region using a syringe needle according to previously reported protocols (Balassiano et al., 2001). Eggs were incubated at 99°F for 3 days after which the shell was removed carefully to expose the CAM and observed for changes in the vascularization pattern. Each treatment condition was performed in triplicates.

### Statistical Analysis

Data are represented as mean and SE from experiments, each performed in triplicate. Statistical significance was evaluated by a two-sided Student’s *t*-test. A value of *P* < 0.05 was considered significant.

## Results

### Enrichment and Characterization of Breast CSCs Showed a Signature Pattern of CSC Markers

**FIGURE 1:**
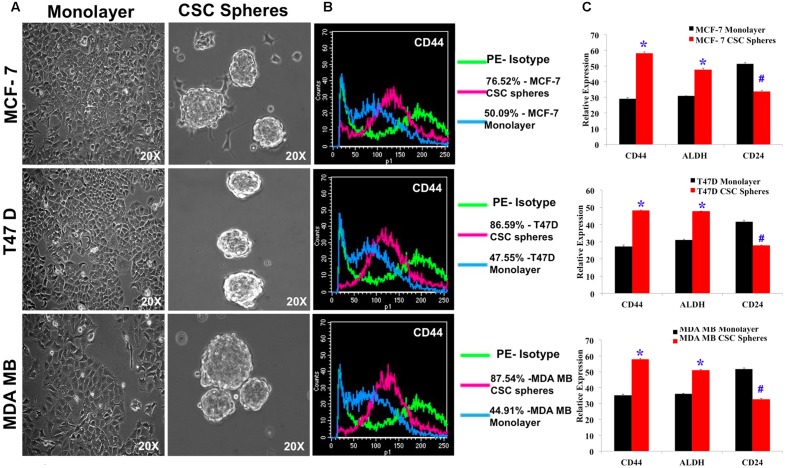
**Characterization of breast CSC signature markers in spheroid cultures of MCF7, T47D and MDA-MB-231 by flow cytometry and quantitative RT-PCR.** **(A)** Photomicrographs of monolayer culture and spheroid cultures grown in CSC medium. **(B)** Flow cytometry study of CD44 positive cells in monolayer culture and spheroid cultures. **(C)** Quantitative RTPCR of the mRNA expression of breast CSC markers, CD44, ALDH and CD24 of MCF7, T47D and MDA-MB-231 cell lines grown in CSC medium. Results are the mean ± SD of three independent experiments performed in triplicates (^∗^*p*-value < 0.05, #*p*-value < 0.01, *n* = 3).

We enriched the CSC population in MCF7, MDA-MB-231 and T47D cell lines by culturing them in serum free, growth factor enriched culture medium and obtained spheroid colonies (**Figure 1A**). The resultant mammospheres were then characterized for the CSC marker CD44 by flow cytometry and the CSC enrichment was determined by comparing the extent of CSC marker expression to the non-CSC monolayer culture for all the three cell lines. MCF7, T47D and MDA-MB-231 cells had an increase of 25, 39, 42% of CD44 expression respectively over monolayer culture (**Figure 1B**). Next, we confirmed the enrichment of CSC by analyzing for the mRNA expression of not only CD44 but also other breast CSC signature markers, CD24 and ALDH. As expected, CD44 and ALDH expression had nearly doubled whereas the mRNA expression of CD24 had decreased significantly in CSC culture (**Figure 1C**). These cells, thus verified for their CSC properties, were used for further experiments.

### Diosgenin Suppresses the Proliferation of Breast CSCs by Inducing Apoptosis by the Activation of Caspase 3/7 and the Release of ROS

**FIGURE 2:**
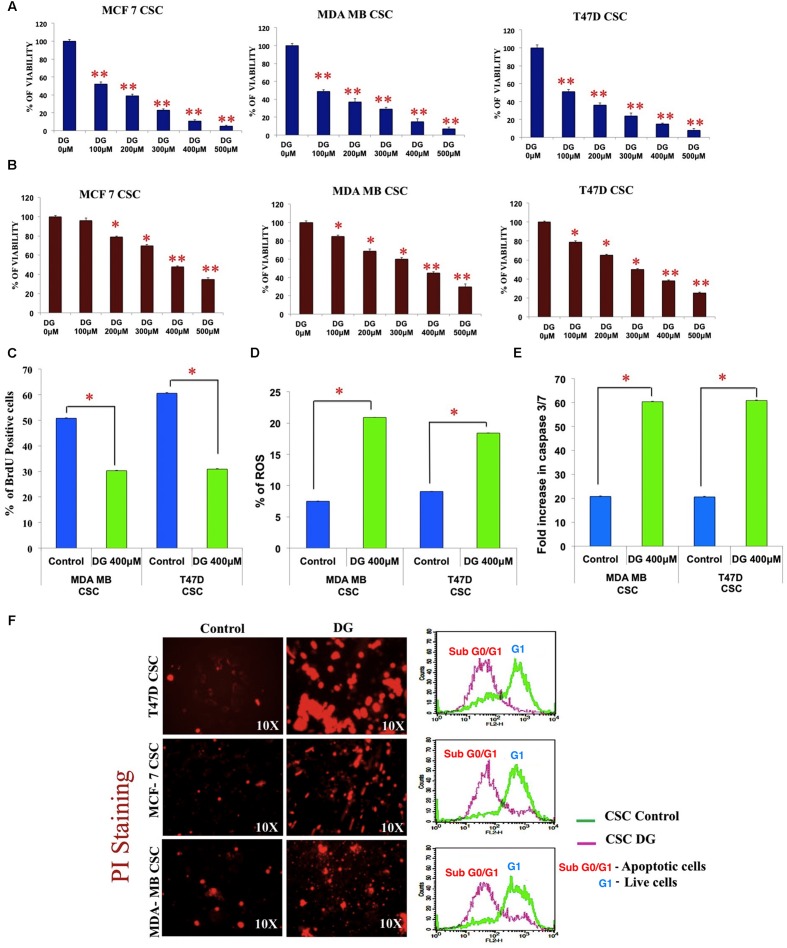
**Inhibition studies of breast CSCs by diosgenin by inhibiting proliferation, inducing apoptosis, release of ROS and cell cycle arrest.** **(A)** MTT assay shows the inhibition pattern of monolayer culture and CSC culture **(B)** of MCF7, T47D and MDA-MB-231 cell lines treated with diosgenin (DG) at various concentrations. DG treatment inhibited proliferation as shown by BrdU assay **(C)**, increased release of ROS **(D)** and elevated levels of caspase 3/7 **(E)**. Data are mean ± SEM. ^∗^*p* < 0.05; ^∗∗^*p* < 0.01, *n* = 3. **(F)** Increased uptake of propidium iodide and accumulation of sub G0 cells of T47D, MCF7 and MDA-MB CSCs after DG treatment is shown by fluorescence microscopy (left panel) and flow cytometry (right panel).

We next checked the effect of DG treatment on monolayer and CSC cells. Using MTT assay, we observed that DG inhibits monolayer cells of MDA-MB 231, T47D and MCF7 cell lines at an IC50 concentration of 100 μM (**Figure 2A**). However, the CSC enriched cells required a much higher concentration of 400 μM for 50% inhibition that further confirmed the chemo-refractory nature of these CSC (**Figure 2B**). The estimated IC50 concentration of 400 μM of DG for CSCs was used for further experiments. After confirming arrest of CSC proliferation by BrdU (**Figure 2C**), we assayed the levels of ROS and caspase 3/7. As shown in **Figure 2D**, DG substantially increased ROS levels in both MDA-MB-231 and T47D CSCs. Caspase 3/7 assay also showed that DG caused a twofold increase suggesting that DG induces caspase-3-dependant apoptosis in bCSCs (**Figure 2E**).

### Drug Intake Intensified and Accumulation of Sub G0/G1 Cells Observed after DG Treatment

The influx of PI, as observed by fluorescence microscopy was much more intense in DG-treated T47D, MCF7 and MDA-MB-231 CSCs, with T47D showing the highest response. Furthermore, flow cytometry after PI labeling revealed an accumulation of cells in the sub G0/G1 phase indicating cell cycle arrest after DG treatment (**Figure 2F**).

### Diosgenin Inhibits CSC-Specific Properties of Mammosphere Formation and CD44 and ALDH Expression

**FIGURE 3:**
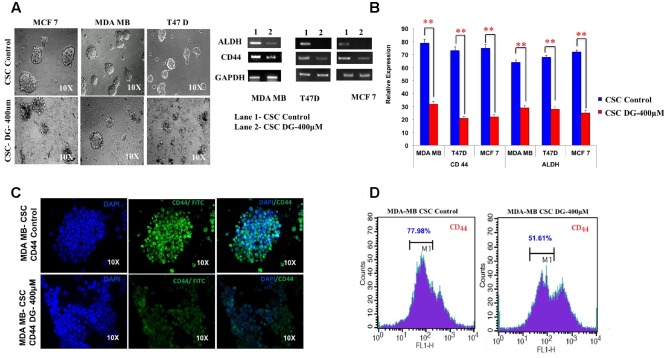
**Diosgenin retards proliferation of breast cancer cells by inhibiting CSC population.** **(A)** Mammosphere disruption assay was performed after treatment with DG on CSCs from MCF-7, MDA-MB-231 and T47D cell lines. **(B)** CD44 CSC marker was analyzed after treatment with DG for 24 h by RTPCR and qRT-CR in these three cell lines. **(C)** Immunocytochemical staining and **(D)** flow cytometry with CD44 of CSCs from MDA-MB-231 showed a decrease in CD44 levels after DG treatment. Data are mean ± SEM. ^∗∗^*p* < 0.01, *n* = 3.

After treatment of bCSC from T47D, MCF7 and MDA-MB-231, we assessed for mammosphere disruption, and saw a marked decrease in the sphere size and disintegration in all the three cell lines (**Figure 3A**). We next investigated the mRNA expression of bCSC markers, CD44 and ALDH, after DG treatment by quantitative RT-PCR. A decrease of expression up to twofold was seen for both CD44 and ALDH (**Figure 3B**). This was further confirmed by CD44-targeted immunocytochemical staining in which the CD44 staining was weak after DG treatment compared to the control bCSCs of MDA-MB cells (**Figure 3C**). We then confirmed this data by flow cytometry which showed about 25% decrease of CD44 positive population after treatment with DG when compared to the control (**Figure 3D**).

### Apoptotic Effect of Diosgenin Is Accompanied by Downregulation of Drug Effluxers, Disruption of the Wnt-β Catenin Pathway

**FIGURE 4:**
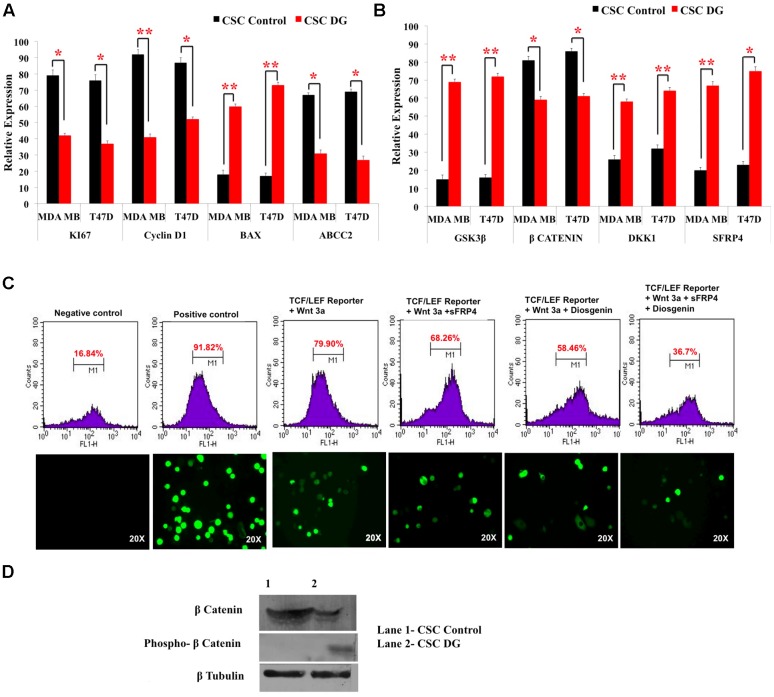
**Downregulation of drug transporters, pro-proliferation markers, upregulation of Wnt antagonists and inhibition of TCF/LEF promoter gene by diosgenin. (A,B)** Gene expression analysis of CSCs from T47D and MDA-MB-231 was done by qRT-PCR of pro-proliferation genes, CyclinD1 and Ki67, apoptotic marker Bax, drug effluxer ABCC2, Wnt antagonists sFRP4, Dkk1, Wnt activator β-catenin and GSK3β kinase. Results are the mean ± SD of three independent experiments performed in triplicates (^∗^*p*-value < 0.05, ^∗∗^*p*-value < 0.01, *n* = 3). **(C)** Transfection of MDA-MB with TCF-LEF promoter showed the expression of GFP reporter regulated by TCF/LEF promoter without Wnt3a stimulation (1), after addition of only Wnt3a (2), Wnt3a and sFRP4 (3), Wnt3a and DG (4), Wnt3a, sFRP4 and DG (5). **(D)** Western blotting revealed increase in phospho β-catenin and decreased β-catenin in DG treated MDA-MB CSCs.

We next examined the expression levels of proliferation-associated markers, Ki67 and cyclin D1 and observed a twofold decrease in bCSCs from T47D and MDA-MB-231 after DG treatment (**Figure 4A**). Consistent with this, pro-apoptotic gene, Bax was increased 2–2.5 fold after DG treatment (**Figure 4A**). As it has been shown that chemoresistance of CSC is associated with overexpression of drug effluxers, we examined for the expression of ABCC2 marker. There was twofold decrease in ABCC2 expression indicating an improved response to DG (**Figure 4A**). Because of the prominent role of Wnt-β catenin signaling in proliferation and in the CSC phenotype (Jang et al., 2015a,b), we examined the expression of Wnt-β catenin pathway related genes. There was a substantial decrease of the key activator β-catenin and nearly threefold increase in GSK3ß which phosphorylates and inactivates β-catenin (**Figure 4B**). Consistent with this finding, there was an overexpression of Wnt antagonists, sFRP4 and Dkk (**Figure 4B**).

### DG Targets Wnt-β Catenin Pathway as Seen by the Inhibition of TCF-LEF Controlled Genes and β Catenin Expression

As we observed a role of the effectors of Wnt-β-catenin pathway at the gene level, we investigated the impact of DG on the promoter, the TCF-LEF regulating Wnt-β-catenin pathway, using the TCF/LEF GFP reporter assay on MDA-MB CSCs. DG treatment reduced GFP expressing cells to 58% after activation with Wnt3a, whereas activation by Wnt3a alone showed 79.9% of GFP positive cells. Addition of the Wnt antagonist sFRP4 also reduced the GFP positive cells to 68.26%, whereas addition of sFRP4 along with DG reduced the GFP population to 36.7%, demonstrating that DG suppresses that Wnt-β-catenin pathway (**Figure 4C**, upper panel). Fluorescence microscopy also revealed the same trend of fewer GFP positive cells after DG treatment and the least number GFP positive cells after DG and sFRP4 treatment (**Figure 4C**, lower panel). To confirm this involvement further, we assessed whether or not β-catenin is inactivated after DG treatment by analyzing the protein levels of active and inactive β-catenin (phospho β-catenin). DG treatment resulted in a marked increase in phospho β-catenin and a decrease in active β-catenin (**Figure 4D**).

### Overexpression of Wnt Antagonist sFRP4 Further Elevates Response of CSCs to DG Accompanied by EMT Reversal

**FIGURE 5:**
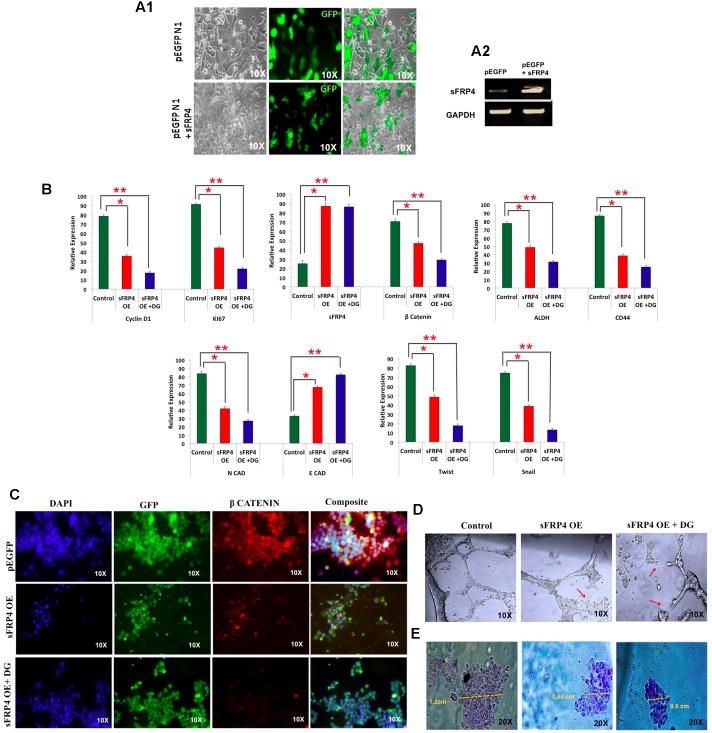
**Breast cancer cells overexpressing Wnt antagonist, sFRP4, is inhibited by diosgenin by suppressing the EMT pathway, retarding cell migration and angiogenesis.** **(A)** Transfection of MDA-MB-231 cells with pEGFP- sFRP4 plasmid showed GFP expression (A1) and overexpression (OE) of sFRP4 (A2). **(B)** qRTPCR analysis of sFRP over-expressing (OE) cells with and without DG treatment for markers for proliferation, Wnt-β-catenin pathway, CSC and EMT markers. **(C)** Immunohistochemistry of β-catenin of sFRP4 OE cells with and without DG treatment. **(D)** Endothelial tube formation assay of sFRP4 OE cells with and without DG treatment showed a marked tube disruption in DG treated cells. **(E)** Cell migration assay of sFRP4 OE cells with and without DG treatment showed reduced migration in DG treated cells. Results are the mean ± SD of three independent experiments performed in triplicates (^∗^*p*-value < 0.05, ^∗∗^*p*-value < 0.01, *n* = 3).

As our results indicated an involvement of Wnt antagonist sFRP4 in DG mediated apoptosis consistent with our earlier findings that sFRP4 addition chemosenstitizes glioma stem cells to drugs (Warrier et al., 2012; Bhuvanalakshmi et al., 2015), we were interested in examining how bCSCs over expressing sFRP4 would respond to DG. After transfecting MDA-MB-231 CSCs with sFRP-EGFP containing plasmid, overexpression was confirmed with the reporter EGFP expression (**Figure 5A1**) and sFRP4 expression (**Figure 5A2**). After treatment of sFRP4-OE cells with DG, we observed OE cells were more responsive to DG and showed a significant decrease in the expression of proliferation markers, Ki67 and CyclinD1 compared to treatment with DG alone (**Figure 5B**). There was a threefold down-regulation, in DG treated OE cells, of pro-invasive genes, Twist and Snail and upregulation of *E*-cadherin and reduction of the EMT marker *N*-cadherin, which was more pronounced in OE cells treated with DG (**Figure 5B**). A similar trend was seen with respect to the CSC markers CD44 and ALDH, with DG treated OE cells showing a more prominent reduction. Expectedly β-catenin expression was also downregulated in DG treated OE cells (**Figure 5B**). It appears that sFRP4 overexpression has sensitized bCSCs to be more susceptible to DG treatment by abrogating pro-invasive and EMT genes which are largely regulated by the Wnt-β-catenin pathway (Loh et al., 2013; Jang et al., 2015b).

### sFRP4 Overexpressed Cells Had Diminished Cell Migration and Angiogenic Tube Formation Ability and Lowered β-Catenin Levels after DG Treatment

There was also a marked loss of β-catenin accumulation in OE cells and further decrease in OE + DG cells as examined by immunocytochemistry (**Figure 5C**). By performing angiogenesis assay, we could see a disruption in ring formation in OE + DG cells (**Figure 5D**). Cell migration assays revealed that migration was impeded in OE and OE + DG cells with a drastic reduction in OE + DG treated cells (**Figure 5E**). This observation is consistent with the downregulation of the pro-invasive and metastatic genes observed in DG treated OE cells.

### RNAi of sFRP4 Reverses the Effect of sFRP4 OE by Increasing Stemness, EMT and Activating the Wnt-β-Catenin Pathway

**FIGURE 6:**
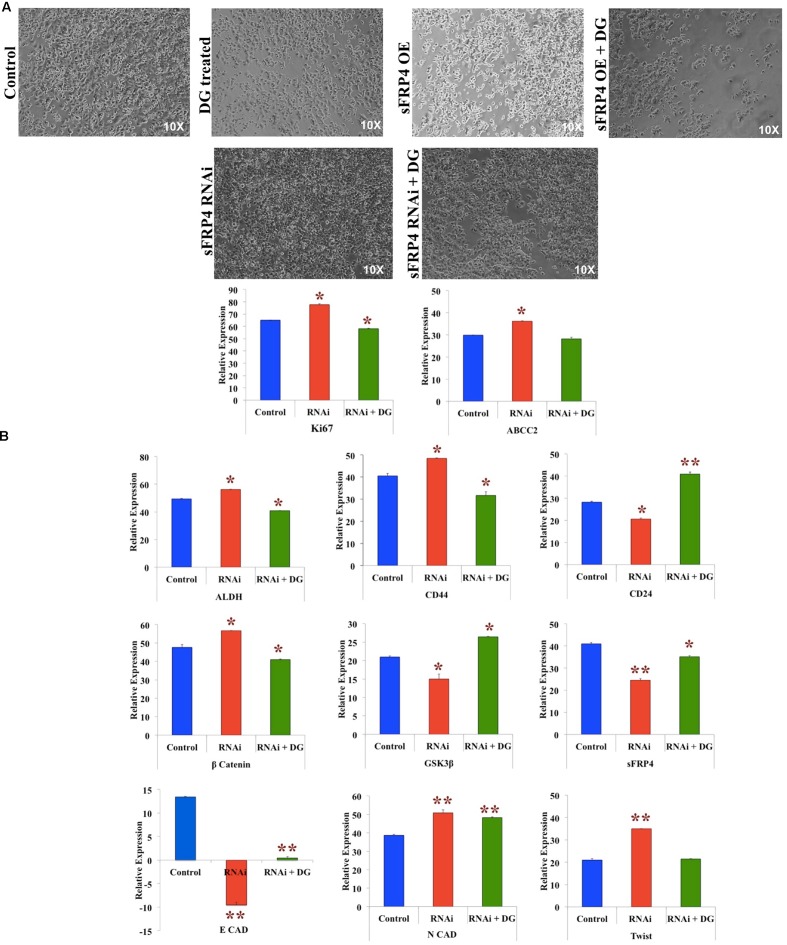
**RNAi of sFRP4 reverses the effect of sFRP4 OE by increasing stemness, inducing EMT and activating the Wnt-β-catenin pathway.** **(A)** Phase contrast photomicrographs of MDA-MB-231 cells with and without DG treatment after sFRP overexpression (OE) and knockdown (RNAi). **(B)** Quantitative RT-PCR of MDA-MB without and with DG treatment, with and without RNAi for proliferation markers and drug effluxer, CSC markers, effectors of the Wnt-β-catenin pathway and EMT pathway. Error bars represent the mean ± SD of three independent experiments performed in triplicates (^∗^*p*-value < 0.05, ^∗∗^*p*-value < 0.01, *n* = 3).

To investigate the effect of depletion of sFRP4, we used RNAi-mediated sFRP4 knockdown in MDA-MB-231 cells and studied if the effect of sFRP OE was reverted. We observed that whilst sFRP4 with DG addition resulted in the maximum cell death, surprisingly knockdown of sFRP4 induced hyperproliferation and sFRP-KD cells had poor response to DG when compared to sFRP-OE with DG (**Figure 6A**). After sFRP-KD, CSC markers CD44, ALDH was upregulated and CD24 was downregulated (**Figure 6B**). Expectedly, β-catenin levels increased and GSK3β and sFRP4 levels increased and treatment of sFRP4-KO with DG reversed these changes so that it was comparable to the control (**Figure 6B**). Unexpectedly, we observed a complete loss of the epithelial marker *E*-cadherin in sFRP4-KO cells. Decrease in *E*-cadherin in sFRP1 KO kidney cells has been previously documented (Ren et al., 2013). The mesenchymal marker *N*-cadherin and pro-invasive marker Twist was increased in sFRP4-KO cells which decreased to control levels after DG treatment (**Figure 6B**).

### Diosgenin Inhibited Vascularization by CSCs *In vivo* Using CAM Assay

**FIGURE 7:**
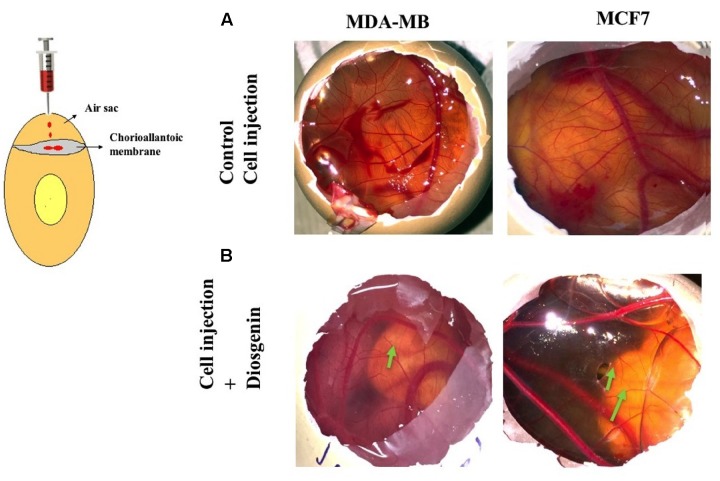
**Diosgenin inhibits vascularization by CSCs *in vivo* using CAM assay.** Angiogenic patterns on the allantoic membrane of chick after injection with untreated CSCs of MDA-MB-231 and MCF7 **(A)** and DG treated CSCs of MDA-MB-231 and MCF7 **(B)**. Arrows indicate the disrupted and weak vasculature after injection with drug treated CSC. Images are representative of experiments performed in triplicates.

The chick embryo provides a good model for the study of tumor-induced angiogenesis (Scanlon et al., 2013). We induced angiogenesis using CSCs of MDA-MB-231, T47D and MCF7 in day’s 4–5 chick eggs with or without treatment with DG. As observed DG treated CSCs had disrupted vasculature, as indicated by arrows (**Figure 7B**) when compared to the excessive vasculature in untreated CSC (**Figure 7A**). This demonstrates the effect of DG in reducing the tumor angiogenic potential of CSCs *in vivo*.

## Discussion

In this work, we identified an inhibitory property of DG on the chemorefractive CSCs which could be mediated by the dysregulation of the Wnt-β-catenin pathway. CSCs represent an important landmark for understanding the mechanisms of chemoresistance and cancer relapse. Breast CSCs are responsible for metastasis and are the seeds that mediate the spread of the tumor. Most of the existing chemotherapeutics, whilst disseminating the non-CSCs, enrich the chemorefractory CSCs. Natural/dietary compounds have an obvious advantage over synthetic chemotherapeutics by virtue of their non-toxic propensity. The advantage of using DG is that it is a phytoestrogen present in a variety of the plants such wild yam, fenugreek and in medicinal plants belonging to the *Dioscorea* species (Yi et al., 2014). In our study, we observed that the defining phenotype of bCSC, namely mammosphere formation and the expression of bCSC markers, CD44 and ALDH, were suppressed by DG in bCSC from three different cell lines, MDA-MB-231, T47D and MCF7, showing differential receptor expression of ER/PR/HER2. This is the first study reporting that DG, one of the biologically active constituent of fenugreek seeds (Shabana et al., 2009), has a direct correlation to the expression of stemness markers. DG has been shown to have an anti cancer effect in many cancers, including breast cancer (Srinivasan et al., 2009), prostate (Chen et al., 2011) and lung (Yan et al., 2009). We observed that the normally chemo resilient bCSCs, were specifically inhibited by DG and these DG treated CSC failed to form robust vasculature *in vivo* in a CAM model. We found that this suppression of growth was accompanied by ROS release which corroborates a similar effect of DG seen on HepG2 cells (Kim et al., 2012). Breast CSCs are known to be sustained by lower concentrations of ROS (Diehn et al., 2009) which is related to an increased expression of free radical scavenger systems. Increased release of ROS deactivates the DNA repair-linked effectors which are at a higher level in breast CSCs (Blanpain et al., 2011). The inhibition of proliferation was due to apoptosis as seen by the marked accumulation of caspase 3/7, indicating that caspase 3 dependent apoptosis was initiated, probably resulting in the activation of Bcl-2 family of proteins associated with intrinsic apoptosis (Boland et al., 2013). Activation of caspase has also been demonstrated in C3A hepatocarcinoma cells after DG treatment (Li et al., 2010).

In this study, we observed the dysregulation of the Wnt pathway at various levels. Wnt target gene, CycD1was downregulated drastically and in contrast Wnt antagonist Dkk1 and sFRP4 were upregulated. Dkk being an inhibitor of the co- receptor of LRP is an antagonist of the Wnt canonical pathway (Bao et al., 2012). sFRP4, on the other hand, is a more versatile inhibitor of the Wnt pathway, being able to block both Wnt (Uren et al., 2000) and Fzd (Dufourcq et al., 2008), thereby effectively grid locking the Wnt-Fzd interactions and blocking the transmission of the downstream signals in the canonical pathway. sFRP4 also plays a role in the non-canonical pathways because of its unique ability to bind both the ligand and receptor of the Wnt pathway. However, the mechanism of action in the non-canonical axis is still rudimentary. We saw an upregulation of both the antagonists after DG treatment. Antagonists of Wnt such as small molecule inhibitor, CWP232228, which inhibits the binding of β-catenin to the TCF promoter (Jang et al., 2015a) or short hairpin RNAs (shRNAs) suppressing Wnt1 expression (Jang et al., 2015b) has been used for targeting breast CSCs. Some natural compounds for e.g., silibinin, have a natural affinity to bind β-catenin and thereby inhibit its downstream activity (Wu et al., 2013). Because of the complexity of action of sFRP4 and because of our earlier observation of targeted inhibition of glioma and head and neck CSCs by sFRP4 (Warrier et al., 2012, 2014; Bhuvanalakshmi et al., 2015), we decided to investigate further a combination effect of DG with sFRP4. Along with the upregulation of Wnt antagonists, GSK3β was OE, suggesting that the phosphorylation and inactivation of β-catenin was initiated. This line of thought *was* reinforced by the observation of higher phospho β-catenin and lower β-catenin proteins in DG treated bCSCs. At the nuclear level, activated β-catenin/Tcf transcriptional activities using reporter assays provides direct evidence for the control of Wnt- β-catenin related genes. Thus, in our study, a confirmatory evidence of DG affecting Wnt- β-catenin regulated machinery was emphatically established when we observed that the Wnt target genes under the control of the TCF/LEF promoter was suppressed after DG treatment. In addition, sFRP4 had an additive effect with DG in the suppression of TCF/LEF promoter suggesting that DG indeed exerts in its effect via the Wnt-β-catenin axis. Similar effect of the arrest of β-catenin-induced transactivation of TCF/LEF has been reported in another widely studied anti-cancer natural compound, curcumin, in colon cancer cells (Jaiswal et al., 2002).

Because of a clear role of Wnt antagonism that we observed in response to DG treatment, we studied a model of breast CSC which was overexpressing sFRP4. Expectedly, the response to DG in sFRP4 OE cells was more drastic wherein we could see the reversal of the EMT. Upregulation of epithelial marker *e*-cadherin was accompanied by a decrease in *N*-cadherin and characteristic EMT markers, twist and snail. We have shown previously in glioma and head and neck CSCs that sFRP4 inhibits the mesenchymal traits and promotes not only epithelial markers but also morphology (Warrier et al., 2014; Bhuvanalakshmi et al., 2015). Probably loss of β-catenin levels that we observed, contributed to attaining epithelial properties after DG treatment. Furthermore, the migratory property was severely hampered in these cells after DG treatment. Cell migration is a pointer toward the metastatic nature of the tumor cells (Wan et al., 2013). Additionally, we could also see that angiogenic ring formation was inhibited in sFRP OE cells after subjecting to DG treatment. These findings endorse earlier reports of sFRP4 having anti-angiogenic properties (Muley et al., 2010) and sFRP4 being able to effectively stall angiogenesis in combination with chemotherapeutics in glioma (Bhuvanalakshmi et al., 2015) and head and neck cancers (Warrier et al., 2014).

Conversely, we show that knockdown of sFRP4 reverses the effect of overexpression. The impact of sFRP4 knockdown on enhancing cellular proliferation is emphatic, implying this Wnt antagonist probably works on pathways directly affecting self renewal. This is line with earlier reports on sFRP1, downregulation of which is associated with poor prognosis and therapeutic response in breast cancers (Klopocki et al., 2004; Veeck et al., 2006). Positive breast CSC markers, CD44 and ALDH also concomitantly increased after sFRP4 knockdown and decreased considerably after DG treatment but a pronounced effect was seen in the negative CSC marker CD24 which increased in sFRP4 knockdown cells treated with DG. CD24 has been shown to enhance DNA damage induced apoptosis in breast cancers (Ju et al., 2011). A similar function maybe attributed to the overexpression of CD24 in DG treated sFRP knockdown cells. Additionally, a clear involvement of sFRP4 after DG treatment is further corroborated by the increase in its expression in sFRP4 knockdown treated with DG. Expression pattern of β-catenin was reverse of the sFRP4 OE cells suggesting a possible link between sFRP4 and the gene expression of β-catenin itself. Unexpectedly, sFRP4 knockdown had a pronounced effect on the expression of *E*-cadherin by completely abrogating its expression which was not restored even after DG treatment. The role of *e*-cadherin in governing a CSC phenotype has not been clearly eludicated. Recent reports indicate a correlation between nuclear localization of β-catenin which in turn regulates the CSC phenotype (Su et al., 2015). However, no direct link has been established between a Wnt antagonist and *e*-cadherin expression. Complete loss of *E*-cadherin seen in sFRP4 knockdown cells indicates a prominent role of this Wnt antagonist in maintaining of the epithelial trait. It is also possible that sFRP4 acts via multiple pathways which need to be explored further.

## Conclusion

Taken together, our study reveals that DG, a steroidal saponin derived from natural compounds, can destroy the highly chemorefractory and pro-metastatic breast cancer stem like cells at higher concentrations. DG, unlike synthetic chemotherapeutics, has little or no toxicity on vital organs in mice (Srinivasan et al., 2009). Further, our analysis also provides a deeper insight into the mode of action of DG at the molecular level. We find a strong role of the Wnt-β-catenin signaling which is interrelated with the expression of stemness traits such as self- renewal and drug resistance. The key traits of CSC contributing to aggressiveness and invasion, such as the EMT and pro-invasive markers, were abrogated by DG treatment in the cell model overexpressing Wnt antagonist. In conclusion, DG could be promoted as a novel therapy targeting bCSCs disabling tumor recurrence, metastasis and relapse and improve patient survival.

## Author Contributions

GB: Performing experiments, preparation of manuscript. B: Preparation of manuscript. KR: Preparation of manuscript. APK: Partial funding of the manuscript, preparation of manuscript. AD: Partial funding of the work, preparation of manuscript. GS: Experimental design, preparation of manuscript. SW: Conceptualization of the study, Procuring funds for the study, experimental design, preparation of manuscript.

## Conflict of Interest Statement

The authors declare that the research was conducted in the absence of any commercial or financial relationships that could be construed as a potential conflict of interest.
